# Meta-analyses of differences in blended and traditional learning outcomes and students' attitudes

**DOI:** 10.3389/fpsyg.2022.926947

**Published:** 2022-09-16

**Authors:** Zhonggen Yu, Wei XU, Paisan Sukjairungwattana

**Affiliations:** ^1^Department of English Studies, Faculty of Foreign Studies, Beijing Language and Culture University, Beijing, China; ^2^Faculty of Humanities and Social Sciences, City University of Macau, Macau, Macao SAR, China; ^3^Faculty of Liberal Arts, Mahidol University, Salaya, Thailand

**Keywords:** meta-analysis, blended learning outcomes, attitude, effect size, information technology

## Abstract

The sudden outbreak of COVID-19 has made blended learning widely accepted, followed by many studies committed to blended learning outcomes and student attitudes. Few studies have, however, focused on the summarized effect of blended learning. To complement this missing link, this study meta-analytically reviews blended learning outcomes and student attitudes by including 30 peer-reviewed journal articles and 70 effect sizes. It concludes that blended learning outcomes are significantly higher than the traditional learning outcomes with a medium effect size, and learners hold significantly more positive attitudes toward blended learning than traditional learning with a medium effect size. Blended learning may be promising, and information technology scientists may focus on the development of more advanced and effective devices to improve blended learning effectiveness.

## Introduction

### Definitions of blended learning

The sudden outbreak of COVID-19 has made blended learning widely accepted (Yu, 2021a) and it has led first generation scholars to engage in online or blended learning (Mates et al., 2021). Blended learning has been rising as a major component of learning methods, with which various definitions of blended learning have been put forward (Yu, 2015). Blended learning is defined as a mixed teaching approach where both online learning and physical face-to-face learning are integrated assisted with mobile or educational technologies (Yu, 2015). Blended learning could not only facilitate learning outcomes but also fill the gap between online learning and physical classroom-based learning supported by information technologies (Vaughan, 2007). Blended learning could also connect learning to working (Bohle-Carbonell et al., 2013) *via* mobile or information technologies. Therefore, blended learning could involve distributed, decentralized, hybrid, and flexible learning (Leidl et al., 2020).

In general, blended learning cannot be simply summarized as simple blends of several learning methods involving both physical and online learning. Blended learning can integrate multiple components, e.g., learning situations where online, physical and working situations can be included; knowledge acquisition mechanisms, e.g., retention and attrition; affective elements in learning, e.g., motivation, satisfaction, encouragement, and cognitive loads; and participants, e.g., teachers, learners, and designers. It could be a complicated blend including the traditional, the for- and in-actions, and experiential factors (Viebig, 2022).

### Blended learning outcomes

Information technology-assisted blended learning is adopted in every discipline at all levels, which proves effective to promote and improve learning outcomes (Dutton et al., 2002; Zhang and Yu, 2021). Blended learning, a student-centered approach, encourages students to have stronger self-regulation and assume more responsibilities than those who receive traditional face-to-face instruction (Yick et al., 2019). Blended learning requires students to rigidly adhere to academic schedules and to participate in learning activities autonomously (Sun and Rueda, 2012). Strong self-regulation and self-confidence may increase students' engagement time, reduce their dropout rates, and improve their learning outcomes (Liang and Tsai, 2008).

Recent decades have witnessed many studies committed to information technology-assisted learning, which reported that digital tools, online evaluation, and adaptive online courses could improve students' academic achievements (Brodersen and Melluso, 2017). Information technology tools can provide adaptive instruction for individual learners. Blended individualized learning could meet diverse needs of learners and improve academic achievements (Jiménez et al., 2007; Yu, 2021b). blended learning could produce positive learning outcomes in higher education (Sankar et al., 2022). The online and physical information technologies have added benefits to individualized learning by increasing their engagement and intensifying their interest in blended learning (McCarthy et al., 2020). Teachers and students have benefited a lot from this rising blended learning method, and many studies have been committed to the effect of blended learning (Sharpe et al., 2006).

Blended learning combined teachers' instruction with information technologies, which has received great popularity at the elementary educational level (Christensen et al., 2013). Blended learning integrated traditional face-to-face learning with information technology-supported online learning (Pytash and O'Byrne, 2018), which enabled students to have easy access to learning resources wherever and whenever they feel convenient. The mode of blended learning could provide students, especially those who possibly failed the course, with individual learning strategies and protocols (Macaruso et al., 2020). Teachers could also adopt an individualized instruction by delivering knowledge through the information technology-assisted platform when or where they desire, which could bridge the gap between satisfactory learning outcomes and students' actual performance (Shanahan and Lonigan, 2010). The information technologies could immerse learners in academic activities, stimulate their motivation, and improve their learning outcomes (Repetto et al., 2018). Despite this, there are still some contradictory findings regarding blended learning outcomes. An example is that no significant differences are revealed in learners' writing performance between the blog and paper-based writing methods (Ellison and Wu, 2008). Blended learning outcomes are thus worth further examination.

### Student attitudes toward blended learning

Many studies have been devoted to the exploration of student attitudes toward blended learning, most of which reported positive student attitudes. Nursing students receiving blended cardiopulmonary resuscitation (CPR) education held more positive attitudes toward the blended approach CPR than the control group who received traditional education (Moon and Hyun, 2019). Integrated with information technologies, the blended learning approach could provide flexible instructional methods for students of all ages. This individualized instructional method could meet students' various social, academic, and educational needs, enhance their engagement, and improve their attitudes toward blended learning (Arrosagaray et al., 2019).

Students hold positive perceptions and attitudes toward asynchronous blended English grammar learning and instruction assisted with computer tools (Pinto-Llorente et al., 2017). They also positively evaluate blended learning assisted with blogs, wikis, podcasts, and virtual classrooms in a teacher education program at RMIT University, Australia (Robertson, 2008). Furthermore, attitudes toward blended learning such as Person-Centered e-Learning (PCeL) exert an important influence on learning outcomes and motivation of students (Motschnig-Pitrik and Mallich, 2004). Teaching, cognitive, and social presences could also influence students' attitudes toward blended learning according the theory of Community of Inquiry (Yin and Yuan, 2022).

Students generally hold positive attitudes toward information technologies to supplement online learning and blended learning (Yu and Yu, 2019; Yu et al., 2019). Attitudes can play an important role in the acceptance of blended learning. Positive attitudes toward blended learning drive students to accept the instruction integrating information technology-assisted instruction with a face-to-face approach, where competitiveness outweighs cooperation among undergraduates (Hwang and Arbaugh, 2009). Despite the closure of educational institutes, most dental students positively evaluate the virtual learning (Cho and Ganesh, 2022). Attitudes toward blended learning are, however, inconsistent in blended physical education (Tenison and Touger-Decker, 2018). Sport and exercise students prefer the traditional face-to-face education despite the pandemic (Finlay et al., 2022). It is thus necessary to explore student attitudes toward blended learning.

### Meta-analytical review studies on blended learning

Numerous studies have been committed to blended learning *via* meta-analyses. It is analytically reviewed that nursing students' knowledge and skills *via* blended learning improve significantly more than traditional learning (Li et al., 2019). Although blended learning is generally effective, the effectiveness depends on the learning contexts and the way of the use of information technologies. The technology use also negatively influences blended learning since there are still negative effect sizes. Motivation, attitudes, and warrant consideration may be important factors influencing the blended learning effect (Mahmud, 2018).

Blended learning could lead to significantly better academic achievements than traditional learning in the discipline of health (Liu et al., 2016). Assisted with computer technologies, blended learning could enhance academic achievements in higher education (Bernard et al., 2014). Blended learning could lead to significantly higher academic achievements among STEM-disciplined students than traditional learning (Vo et al., 2017). Furthermore, blended learning could improve interactions and critical thinking abilities (Chang and Yeh, 2021), conducive to positive learning outcomes (Means et al., 2013).

Although many studies have systematically and meta-analytically reviewed the effect of blended learning, few of them have summarized the learning outcomes of and attitudes toward blended learning. Except for overall student attitudes toward blended learning, this meta-analytical review will examine the overall effect of blended learning on learning outcomes. Learning outcomes in this study include English listening, speaking skills, and critical thinking, encouragement of computer science students, student engagement, self-efficacy, motivation, knowledge, online achievement test scores, and writing skills.

## Research questions and hypotheses

We proposed two research questions, i.e., (1) What differences in learning outcomes can be established between blended learning and the traditional face-to-face approach? (2) What differences in learner attitudes can be established between blended learning and the traditional face-to-face approach? Research hypotheses tend to be proposed and tested in meta-analyses in the field of education (e.g., Foster, 2021; Katsarov et al., 2021). Thus, we proposed two alternative research hypotheses based on the research questions, i.e., (1) Blended learning outcomes are significantly higher than the traditional learning outcomes; (2) Learners hold significantly more positive attitudes toward blended learning than traditional learning.

## Research methods

### Literature search

**Figure 1:**
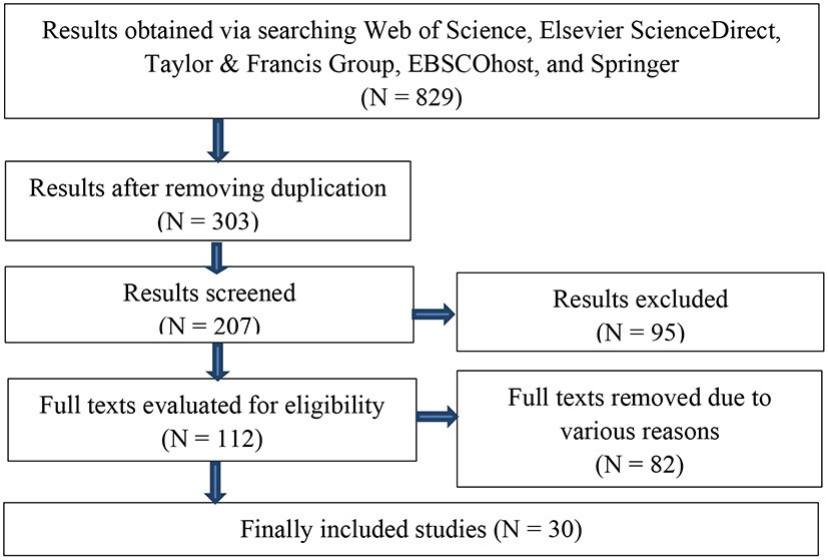
A flowchart of literature search.

The literature search a systematic literature review based on a strict protocol. We followed a flowchart to search literature (Figure 1). EBSCOhost includes many databases such as Business Source Premier, Newspaper Source, Education Resources Information Center (ERIC), MEDLINE, Regional Business News, Library, Information Science & Technology Abstracts, GreenFILE, Teacher Reference Center, European Views of the Americas: 1493 to 1750, eBook Collection (EBSCOhost), EBSCO eClassics Collection (EBSCOhost), OpenDissertations, Modern Language Association (MLA) Directory of Periodicals, MLA International Bibliography with Full Text, Academic Search Complete, Business Source Complete, and Political Science Complete.

*Via* Boolean logic/phrase, two independent researchers, experienced lecturers in the research team, entered *TI blended learning OR hybrid learning* (control OR treatment OR experimental) into EBSCOhost for academic search ranging from 2000 to 2020, and 508 results returned (25 August 2020). They reduced the results to 137 by limiting them to *fulltext, academic peer-reviewed journal, academic theoretical journal, journal*, and *English*. Finally, they selected 4 peer-reviewed articles of high quality.

Additionally, they obtained 52 results by searching Springer *via* “(control OR treatment OR experimental)” AND TI “blended learning OR hybrid learning” within the field of Educational Technology, ranging from 2000 to 2020. After removing 47 results, we selected 5 articles of high quality. We obtained 8 results from Web of Science (WOS) and selected 4 of them.

By keying in [Publication Title: blended learning OR hybrid learning] AND [[All: control] OR [All: treatment] OR [All: experimental]] in Taylor & Francis, they obtained 205 results, ranging from 2000 to 2020. By selecting the subject “education”, we reduced the results to 142. They selected 6 high-quality peer-reviewed articles after perusing the articles.

By keying in “Title abstract, keywords: control OR treatment OR experimental, and Title: blended learning OR hybrid learning” in the database Elsevier ScienceDirect, they obtained 56 results, ranging from 2000 to 2020. After careful reading and screening, they selected 11 of them. Besides, we selected results based on both inclusion and exclusion criteria, as well as The STARLITE tool (Booth, 2006).

### Inclusion criteria

The studies will be considered eligible if they: (1) focus on blended learning effect *via* controlled and randomized comparative design; (2) divide the participants into both control and treatment groups and the control group receives traditional face-to-face learning, while the treatment group receives blended learning; (3) include student attitudes toward blended learning or blended learning outcomes such as test scores, satisfaction, and conceptual understanding; (4) are published in English.; (5) belong to peer-reviewed journal articles.

### Exclusion criteria

The studies will be excluded if they: (1) are not related to the comparison between blended and traditional learning; (2) are conference abstracts, editorial articles, review articles or meta-analyses; (3) focus on online learning technologies rather than the use of them in education; (4) cannot provide enough information for meta-analysis even after we correspond with the authors.

### Evaluation of the literature

We also used University of West England Framework for Critically Appraising Research Articles (Moule et al., 2003) to evaluate the quality of literature. The star rating system used in the evaluation of the literature includes criteria evaluating different sections of a target paper. For the introduction part, two questions are raised to evaluate the quality, i.e., “Is there a clear statement about the topic being investigated? Is there a clear rationale for the research?” Several questions are formulated to evaluate the quality of the method section including clarity, data collection and analysis, qualitative or quantitative research methods. The paper is also evaluated in terms of an ethical statement. For the results/discussion section, we focus on both quantitative and qualitative explorations. The conclusion section needs to include acknowledgment and recommendations.

We deleted those of lower quality and adopted those of higher quality. The quality was rated with “star” (Ren et al., 2019). The article rated higher than nine stars was considered high-quality literature, seven to eight stars indicating medium quality and less than seven stars indicating lower quality. Different opinions were discussed and decided by a third researcher. Finally, we selected 30 peer-reviewed journal articles (Table 1) from the four online databases.

**Table 1:** Included literature for meta-analysis.

**N**	**Author/year**	**Sample size**	**Journal**	**Source**	**Focus**
		**T/C**			
1	Yang (2012)	54/54	Computer Assisted Language Learning	Taylor & Francis	Reading progress
2	Monteiro and Morrison (2014)	24/42	Educational Research and Evaluation	Taylor & Francis	Time spent
3	Mueller et al. (2020)	24/91	International Journal of Research & Method in Education	Taylor & Francis	Course grades
4	Yick et al. (2019)	49/49	International Journal of Fashion Design, Technology and Education	Taylor & Francis	Grades in assignment
5	McCarthy et al. (2020)	1707/1702	Journal of Research on Technology in Education	Taylor & Francis	Academic progress
6	Botts et al. (2018)	269/100	PRIMUS	Taylor & Francis	Time spent and problem-solving
7	Al-Qatawneh et al. (2020)	47/47	Education and Information Technologies	Springer	Test scores and attitudes
8	Bazelais and Doleck (2018)	27/24	Education and Information Technologies	Springer	Exam scores and knowledge state
9	Lopez-Perez et al. (2013)	598/530	Educational Technology Research and Development	Springer	Exam scores
10	Macaruso et al. (2020)	371/251	Educational Technology Research and Development	Springer	Reading achievements
11	Pérez-Marín and Pascual-Nieto (2012)	64/67	Journal of Science Education and Technology	Springer	Test scores
12	Yang et al. (2013)	83/83	Computers & Education	Elsevier	Critical thinking, listening, and speaking skills
13	Baepler et al. (2014)	218/208	Computers & Education	Elsevier	Learning outcomes
14	Cortizo et al. (2010)	30/30	Computers & Education	Elsevier	Level of knowledge
15	Jia et al. (2012)	47/49	Computers & Education	Elsevier	English test scores
16	Liu et al. (2016)	42/42	Internet and Higher Education	Elsevier	Oral proficiency
17	McCutcheon et al. (2018)	62/60	International Journal of Nursing Studies	Elsevier	Attitude and motivation
18	Olitsky and Cosgrove (2014)	82/236	International Review of Economics Education	Elsevier	Exam scores
19	Shorey et al. (2017)	124/124	Nurse Education Today	Elsevier	Attitude, satisfaction, communication and self-efficacy
20	Thai et al. (2017)	22/22	Computers & Education	Elsevier	Learning performance, self-efficacy, intrinsic motivation, and perceived flexibility
21	Yeh et al. (2011)	40/40	Computers & Education	Elsevier	Knowledge, skill, and disposition improvement
22	Yen and Lee (2011)	5/17	Computers & Education	Elsevier	Online achievement test comprehension and application
23	Chang et al. (2014)	33/32	IRRODL	EBSCOhost	Achievement test scores, self-assessment-cognition, skill, and attitude
24	Zhou (2018)	32/32	Educational Sciences: Theory & Practice	EBSCOhost	Writing content relevance
25	Gordon et al. (2005)	73/73	Medical Teacher	EBSCOhost	Gain in knowledge
26	Hill et al. (2017)	46/46	Behaviour & Information Technology	EBSCOhost	Final exam scores
27	Gong et al. (2021)	100/100	PeerJ	WOS	Examination scores in medical school/overall satisfaction
28	Ma et al. (2021)	55/54	Advances in Physiology Education	WOS	Attitudes toward healthcare and management/test of HSM knowledge
29	Wang et al. (2020)	52/47	International Journal of Mobile and Blended Learning	WOS	GFB knowledge
30	Suana et al. (2019)	30/32	International Journal of Instruction	WOS	Blended learning tests

## Results

The result section develops based on the proposed alternative research hypotheses.

**H1. Blended learning outcomes are significantly higher than the traditional learning outcomes**

**Figure 2:**
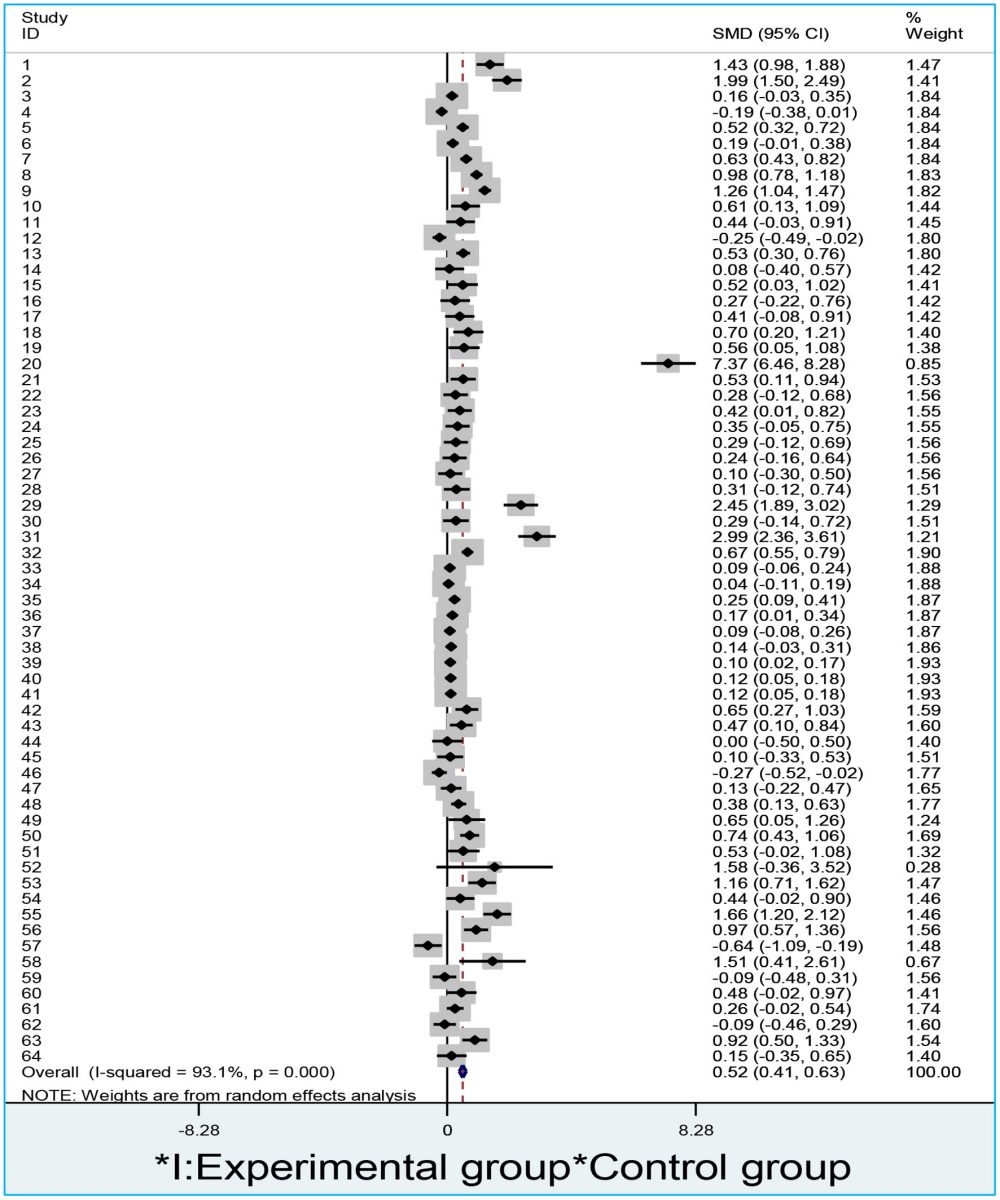
A forest plot of blended vs. traditional learning outcomes.

Figure 2 addresses this research hypothesis. Two researchers co-worked to calculate the mean, mean difference, and confidence intervals on the basis of the research procedure. We obtained in total 64 results from 30 studies. Figure 2 presents the results calculated from 30 studies using Stata/MP 14.0.

If the value of I^2^ is larger than 50%, the between-studies variation will be considered heterogeneous (Ren et al., 2019) and we should switch a fixed-effect to a random-effect model. As shown in Figure 2, the between-studies variation is heterogeneous (I^2^ = 93.1%; *p* < 0.00001), so we apply a random-effect model to the analysis. Means, standard deviations, total numbers of participants and mean differences between blended groups and control groups are indicated, as well as a forest plot on the right. The values of weights indicate the power of an individual study influencing the summarized results. The higher the weight is the more powerful influence an individual study will exert on a summarized result. The studies conducted by Baepler et al. (2014) and Botts et al. (2018) have the highest weight values (3.0%), hence influencing the summarized result to a large extent. By contrast, the studies conducted by Yang (2012), Yang et al. (2013), Monteiro and Morrison (2014), and Shorey et al. (2017) have the lowest value (0%) of weight. Therefore, they exert a minimal influence on the summarized result.

In the middle of the forest plot, the vertical line is referred to as a no-effect line. The result will be considered insignificantly different if the line is touched. The diamond at the bottom of the forest line indicates the summarized result. The former is negatively correlated with the latter. The narrower the diamond is, the stronger confidence the result will have. As shown in Figure 2, the diamond is narrow (*d* = 0.52), ranging from 0.41 to 0.63 in 95% CI, which indicates that the result has a strong confidence. This result does not touch the no-effect line. The diamond is placed on the right side of the no-effect line, indicating the mean of blended learning outcomes is larger than that of the traditional learning. Therefore, we accept the first research hypothesis that the blended learning outcomes are significantly higher than the traditional learning outcomes.

**Figure 3:**
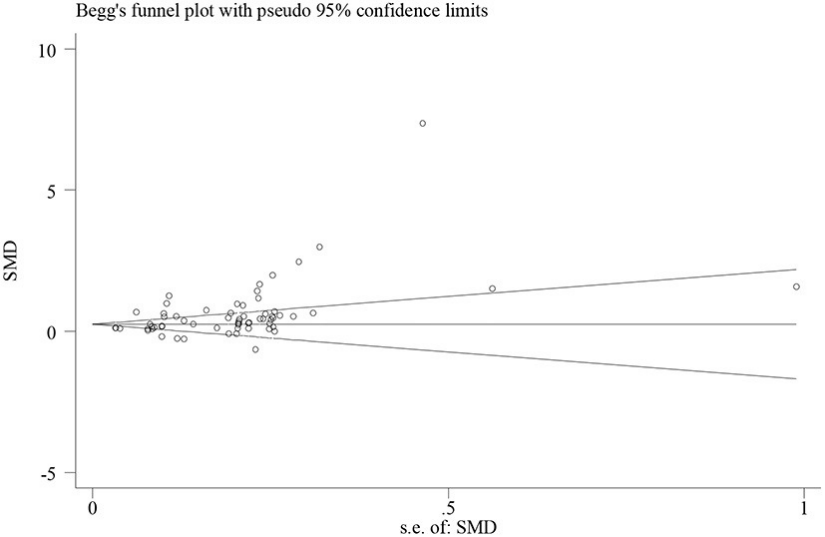
A funnel plot of blended vs. traditional learning outcomes.

**Figure 4:**
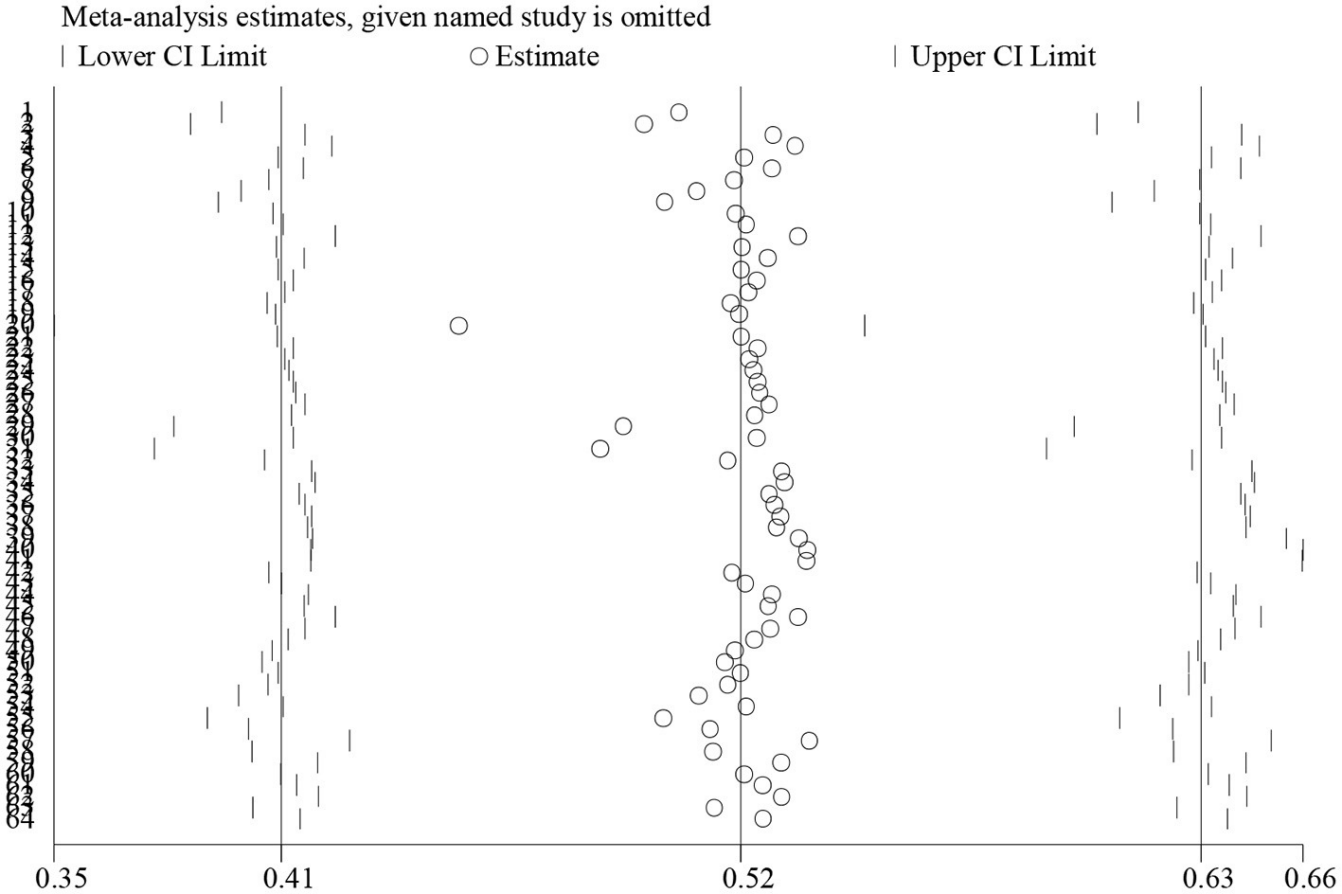
The sensitivity analysis of blended vs. traditional learning outcomes.

However, publication bias may exist. As shown in Figure 3, one dot indicates an individual study. The unbiased distribution of dots is symmetrically aligned on both sides of the middle line. Studies on blended-to-traditional learning outcomes are, however, asymmetrically distributed to the right of the middle line. This indicates that more positive results might have been published than negative ones (Egger's test coefficient = 2.29, *t* = 4.02, *p* = 0.00; Begg's test *z* = 4.02, *p* = 0.00). The sensitivity analysis (Figure 4) shows that no individual study influences the pooled effect size since all of the meta-analysis estimates exist between the lower and upper CI limits given a named study is omitted. This indicates that the results of this meta-analysis are stable.

**H2. Learners hold significantly more positive attitudes toward blended learning than traditional learning**

**Figure 5:**
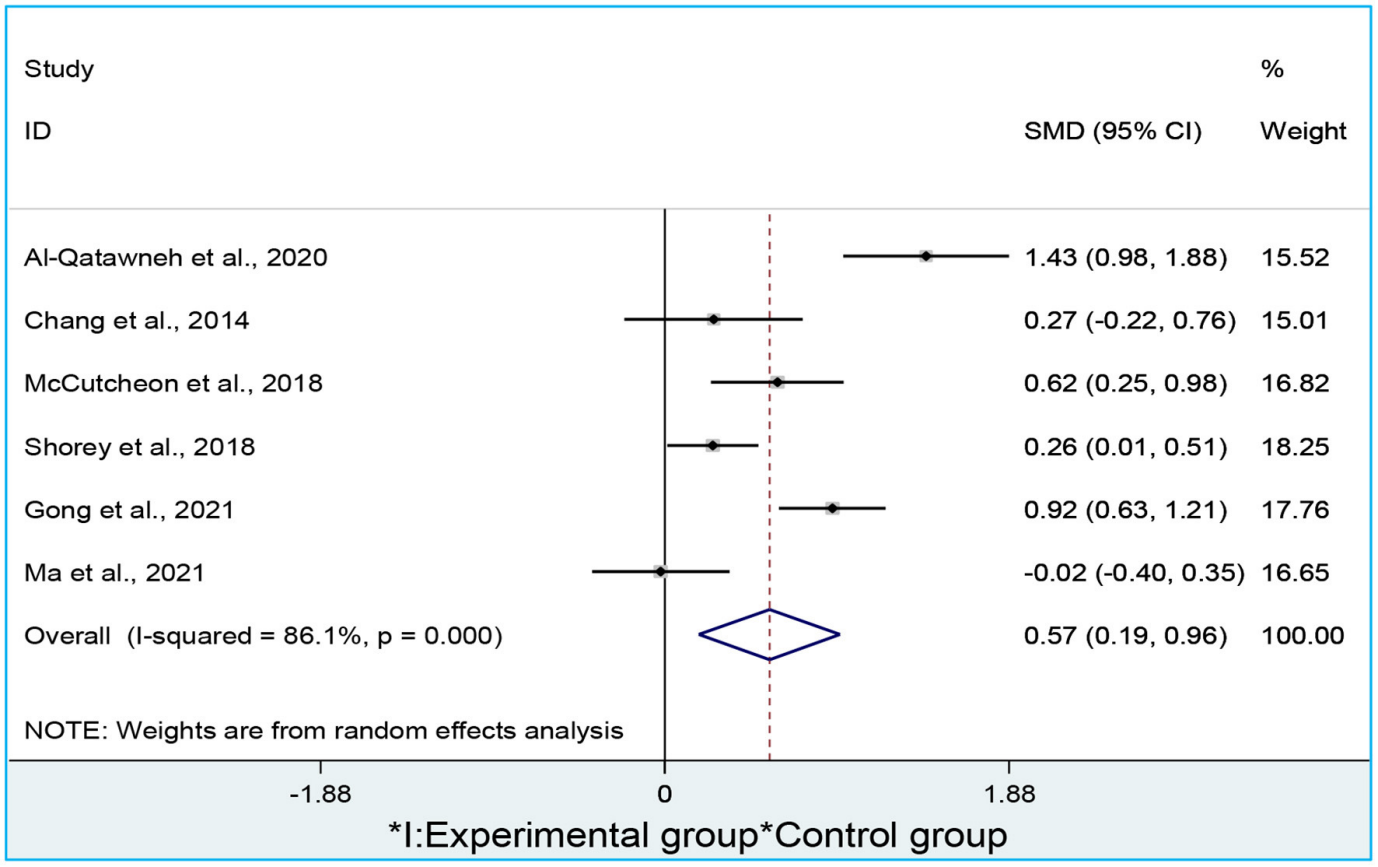
A forest plot of attitudes toward blended learning.

Figure 5 addresses the second research hypothesis. Similarly, both researchers cooperated to calculate the *mean, mean difference*, and *confidence intervals* according to the research design. We finally obtained in total 4 results from 4 studies. Figure 5 presents the results calculated from the 4 studies. We apply a random-effect model to the analysis since the between-studies variation is heterogeneous (I^2^ = 86.1%; *p* = 0.004). We retrieved the data from four studies to address the question, where the data provided by Chang et al. (2014) has the heaviest weight (44.9%), exerting the greatest influence on the summarized result. On the contrary, the study conducted by Shorey et al. (2017) has the lightest weight (0.0%), exerting a minimal influence on the summarized result.

The diamond summarizing the final result is narrow (*d* = 0.57), ranging from 0.19 to 0.96 in 95% CI. This indicates that the summarized result is reliable and convincing. The summarized result does not touch the vertical no-effect line and the result is placed on the right side. This implies that the mean of blended learning is significantly larger than that of the traditional learning. Therefore, we accept the second research hypothesis that learners hold significantly more positive attitudes toward blended learning than traditional learning.

**Figure 6:**
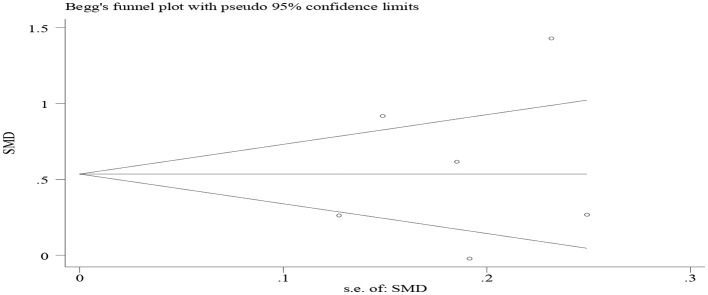
A funnel plot of attitudes toward blended learning.

**Figure 7:**
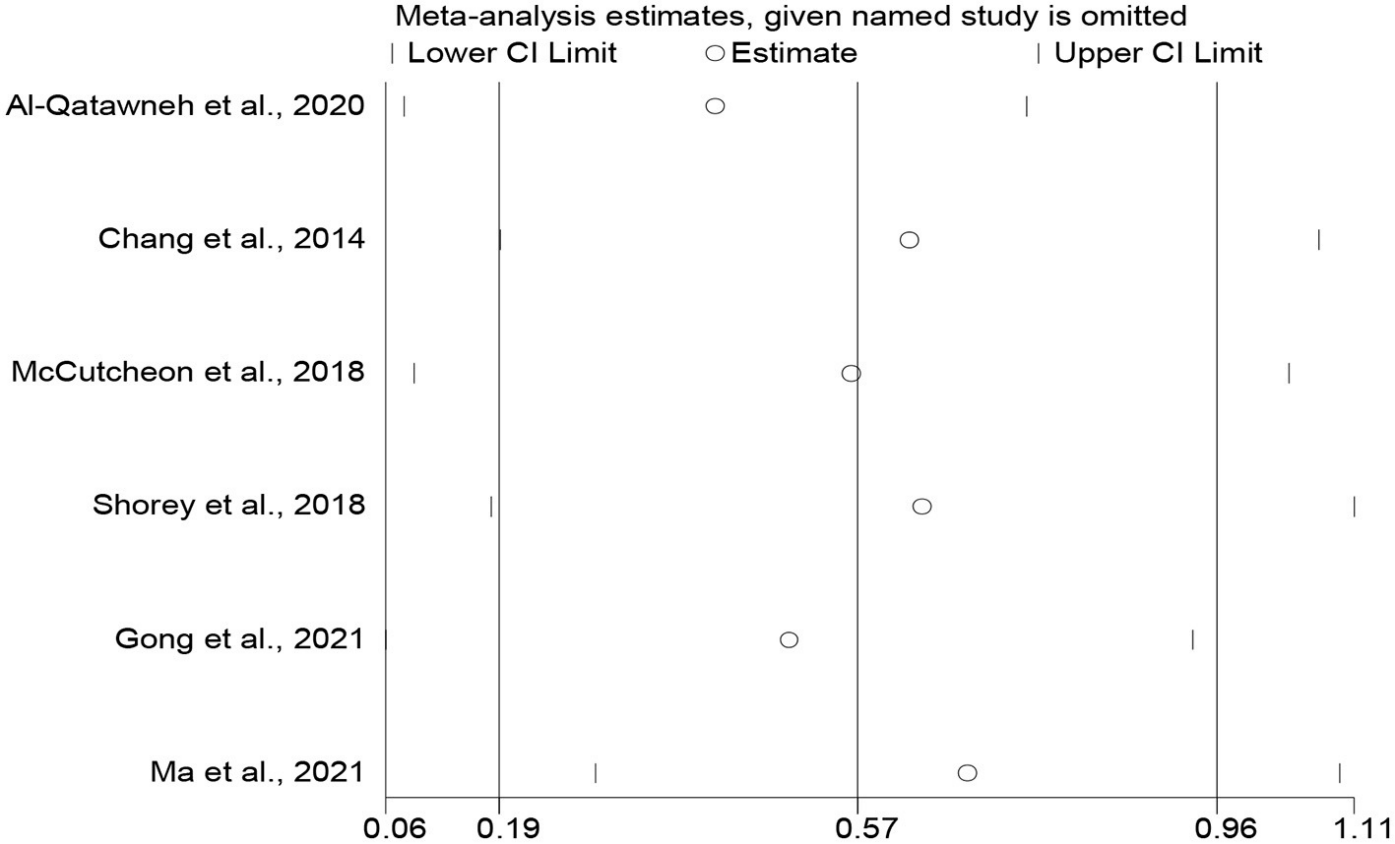
The sensitivity analysis of attitudes toward blended learning.

We did not find publication bias. Studies on attitudes toward blended learning are symmetrically distributed along both sides of the middle line (Figure 6). This means that more positive results may have been published than negative ones (Egger's test coefficient = 2.29, *t* = 0.45, *p* = 0.679, 95% CI = −11.99, 16.58; Begg's test *z* = 0.19, *p* = 0.581). We also carried out a sensitivity analysis (Figure 7), revealing that the meta-analytical results are stable since the meta-analysis estimates are all positioned between the lower and upper CI limits given a name study is omitted.

### Calculation of effect sizes

The Cohen's *d* effect size gains popularity in studies of social sciences. The effect size is considered small if *d* < 0.2, medium if 0.5 < *d* < 0.8, large if *d* > 0.8 (Cohen, 1988). We calculated the effect sizes *via* Cohen's *d* formula. The effect sizes were obtained through the standardized mean differences divided by the pooled standard deviation across both treatment and control groups. We obtained 74 effect sizes from the selected 30 peer-reviewed journal articles (Table 2).

**Table 2:** Effect sizes of blended learning outcomes.

**N**	**Author/year**	**Effect size (*d*)**	**Total**
1	Yang (2012)	0.74 [0.43, 1.06]/0.52 [−0.03, 1.08]/1.26 [−0.75, 3.27]/1.15 [0.70, 1.61]/0.44 [−0.03, 0.90]/1.65 [1.19, 2.11]	6
2	Monteiro and Morrison (2014)	0.00 [−0.50, 0.50]	1
3	Mueller et al. (2020)	0.10 [−0.33, 0.53]	1
4	Yick et al. (2019)	−0.09 [−0.48, 0.31]	1
5	McCarthy et al. (2020)	0.10 [0.02, 0.17]/0.12 [0.05, 0.18]/0.12 [0.05, 0.18]	3
6	Botts et al. (2018)	−0.25 [−0.49, −0.02]/0.53 [0.30, 0.76]	2
7	Al-Qatawneh et al. (2020)	1.42 [0.96, 1.87]/1.98 [1.48, 2.47]	2
8	Bazelais and Doleck (2018)	0.60 [0.13, 1.08]/0.44 [−0.03, 0.91]	2
9	Lopez-Perez et al. (2013)	0.67 [0.55, 0.79]	1
10	Macaruso et al. (2020)	0.09 [−0.06, 0.24]/0.04 [−0.11, 0.19]/0.25 [0.09, 0.41]/0.17 [0.01, 0.34]/0.09 [−0.08, 0.26]/0.14 [−0.03, 0.31]/0.10 [0.02, 0.17]/0.12 [0.05, 0.18]/0.12 [0.05, 0.18]	9
11	Pérez-Marín and Pascual-Nieto (2012)	0.13 [−0.22, 0.47]	1
12	Yang et al. (2013)	0.74 [0.43, 1.06]/0.52 [−0.03, 1.08]/1.26 [−0.75, 3.27]/1.15 [0.70, 1.61]/0.44 [−0.03, 0.90]/1.65 [1.19, 2.11]	6
13	Baepler et al. (2014)	0.16 [−0.03, 0.35]/−0.18 [−0.38, 0.01]/0.52 [0.32, 0.71]/0.18 [−0.01, 0.38]/0.63 [0.43, 0.82]/0.98 [0.77, 1.18]/1.25 [1.04, 1.46]	7
14	Cortizo et al. (2010)	0.56 [0.04, 1.07]	1
15	Jia et al. (2012)	0.28 [−0.12, 0.68]/0.41 [0.01, 0.82]/0.35 [−0.06, 0.75]/0.28 [−0.12, 0.69]/0.24 [−0.16, 0.64]/0.10 [−0.30, 0.50]	6
16	Liu et al. (2016)	0.31 [−0.12, 0.74]/2.43 [1.86, 3.00]/0.28 [−0.15, 0.71]/2.96 [2.33, 3.59]	4
17	McCutcheon et al. (2018)	0.65 [0.27, 1.03]/0.47 [0.09, 0.84]	2
18	Olitsky and Cosgrove (2014)	−0.27 [−0.52, −0.02]	1
19	Shorey et al. (2017)	0.38 [0.13, 0.63]	1
20	Thai et al. (2017)	0.64 [0.03, 1.25]	1
21	Yeh et al. (2011)	−0.63 [−1.08, −0.18]	1
22	Yen and Lee (2011)	1.45 [0.35, 2.55]	1
23	Chang et al. (2014)	0.08 [−0.41, 0.57]/0.52 [0.02, 1.01]/0.26 [−0.22, 0.75]/0.41 [−0.08, 0.90]/0.70 [0.19, 1.20]	5
24	Zhou (2018)	0.47 [−0.03, 0.97]	1
25	Gordon et al. (2005)	7.33 [6.42, 8.24]	1
26	Hill et al. (2017)	0.52 [0.11, 0.94]	1
**27**	Gong et al. (2021)	0.262[−0.016, 0.541]	1
28	Ma et al. (2021)	−0.086[−0.461, 0.290]	1
29	Wang et al. (2020)	0.916[0.501, 1.331]	1
30	Suana et al. (2019)	0.153[−0.345, 0.652]	1
31	**Total**	**0.522[0.410, 0.634]**	**72**

As for blended learning outcomes, the study of Gordon et al. (2005) obtained a large effect size [*d* = 7.33 (95% CI: 6.42, 8.24)], while the study of Monteiro and Morrison (2014) gained the smallest effect size [*d* = 0.00 (95% CI: −0.5, 0.5)]. Most of the other studies obtained medium effect size, e.g., Baepler et al. (2014) [*d* = 0.52 (95% CI: 0.32, 0.71)], Hill et al. (2017) [*d* = 0.52 (95% CI: 0.11, 0.94)], and Botts et al. (2018) [*d* = 0.53 (95% CI: 0.30, 0.76)]. The total effect size is at the medium level [*d* = 0.522 (95% CI: 0.410, 0.634)].

As for student attitudes toward blended learning, Al-Qatawneh et al. (2020) obtained a large effect size [*d* = 1.42 (95% CI: 0.96, 1.87)], while McCutcheon et al. (2018) obtained a medium effect size [*d* = 0.61 (95% CI: 0.25, 0.98)], and Chang et al. (2014) obtained a small size [*d* = 0.26 (95% CI: −0.22, 0.75)]. So did Shorey et al. (2017) [*d* = 0.26 (95% CI: 0.01, 0.51)]. The total effect size is at the medium level [*d* = 0.63 (95% CI: 0.14, 1.11)] (Table 3).

**Table 3:** Effect sizes of attitudes toward blended learning.

**N**	**Author/year**	**Effect size (*d*)**
1	Al-Qatawneh et al. (2020)	1.42 [0.96, 1.87]
2	Chang et al. (2014)	0.26 [−0.22, 0.75]
3	McCutcheon et al. (2018)	0.61 [0.25, 0.98]
4	Shorey et al. (2017)	0.26 [0.01, 0.51]
5	Gong et al. (2021)	0.917 [0.626, 1.209]
6	Ma et al. (2021)	−0.022 [−0.397, 0.354]
7	Total	0.63 [0.14, 1.11]

## Discussion

Blended learning, an approach gaining high popularity, has also been categorized into the “third generation” of information technology-aided education (Phipps and Merisotis, 1999, p. 26). The first generation means correspondence education *via* a one-way pedagogical approach, which adopts traditional tools such as radio, television, broadcaster, and mail. The second generation develops into distance education with the help of traditional instructional technologies, e.g., multimedia projecting systems and computer software assisted boards. The third generation, conducive to higher education (Phipps and Merisotis, 1999, p. 26), evolves into an advanced stage, featuring various blends of information technologies, face-to-face learning methods, opinion sharing platforms, communicative platforms, and discussion forums. This third generation takes advantage of many benefits of the information technologies and optimizes the advantages of various instructional approaches. It is, therefore, reasonable to find that blended learning can lead to higher learning outcomes than traditional learning.

Blended learning can offset the disadvantages of online learning and traditional face-to-face learning. Online learning cannot be applied to all kinds of courses. Some of them may be appropriate for traditional learning, while others may obtain benefits from online learning. The mode of instruction may vary on the basis of instructional purposes of the specific course (Bolliger and Martindale, 2004). Blended learning combines online with traditional face-to-face learning and thus compensates for the disadvantages and expands the advantages (Bersin, 2004). Online learning may cause lower engagement and higher dropout rates, while the traditional learning may give rise to inconvenience in time and space of instruction. Blended learning can minimize these drawbacks by flexibly arranging learning activities and frequently encouraging students to engage in learning (Singh, 2003). Blended learning can also cater for different learners accustomed to various learning styles, which has led blended learning to be widely accepted (Bonk, 2006). This individualized feature of blended learning has enabled it to be a tendency of the pedagogical approach, which may also have caused a positive student attitude toward blended learning.

Blended with information technologies such as blog, twitter, Facebook, and messenger, learners may feel free and easy to express their opinions because keying in the discussion forum is more relaxing and motivating than paper-based writing (Kitchakarn, 2012). Assisted with blogs in writing, learners may improve their reflection compared with the traditional writing on printed paper (Harland and Wondra, 2011). These findings may account for why participants hold positive attitudes toward blog-based writing rather than paper-based writing (Arslan, 2014). Students may hold positive attitudes toward the effectiveness of blogs in learning because they believe they can conveniently access various viewpoints and increase the engagement in writing practice (Ellison and Wu, 2008).

The rationales for favorable attitudes toward blended learning may be of variety in the information age. The blended learning integrates information technologies into learning. Students can obtain a sea of academic resources through their mobile devices conveniently. They can also review what they feel interested in wherever and whenever they feel convenient. They can share opinions with peers and teachers through the application installed in their mobile devices, record their voices *via* recorders, or send messages, voices, or videos to anybody they desire to share. They can also review their rankings *via* the calculation function of the information technology, which may display their learning profile and encourage them to learn.

The information technology-blended approach can also catch learners' attention by displaying colorful pictures or animate videos. Thus, their satisfaction will be enhanced, coupled with favorable attitudes and improved learning outcomes. Blended learning can also facilitate learning effectiveness and speed up their knowledge acquisition, which will potentially cultivate the favorable attitudes. However, excessive exposure to the blended learning approach may bring about distractions, which may explain the phenomenon that blended learning outcomes sometimes remain insignificantly different from traditional learning.

While this study revealed positive blended learning outcomes and students' attitudes by means of meta-analysis and rigid design, the blended process might need further exploration. Given that blended learning can be implemented in several different ways, comparing only outcomes without considering the actual processes that led to those outcomes can be quite problematic. The flipped pedagogical approach has been frequently integrated into the blended learning methods. The lectures are moved out of class that is implemented online before class. The in-class lecturing has been converted to academic activities under the guidance of the lecturer. After class, students can interact with peers and teachers through the online communicative applications (Baepler et al., 2014). Another model of blended methods was implemented, which included five features beneficial to learning and teaching effectiveness (McCarthy et al., 2020). Thus, the outcomes of the study could be enhanced by including the implementation process of blended methods.

## Conclusion

This concluding section will summarize major findings and limitations of this study, together with future research directions.

### Major findings

After meta-analysis and calculation of effect sizes, this study concludes that blended learning outcomes are significantly higher than the traditional learning outcomes with a medium effect size, and learners hold significantly more positive attitudes toward blended learning than traditional learning with a medium effect size.

Findings in this study are generally consistent with previous studies. The blended pedagogical approach could improve nurses' research abilities and critical thinking and lead to positive outcomes (Chen et al., 2021). Blended learning could satisfy the urgent needs of education during the COVID-19 pandemic and in the future (Ahmed and Opoku, 2021). Most of students held positive attitudes toward blended learning and teaching although they desired to change the role of teachers and students, as well as the method of knowledge delivery (Zarrinfard et al., 2021). The blended delivery of knowledge could increase the interactions and learning opportunities among students who could then be motivated and receive flexible education (Bashir et al., 2021).

### Limitations

Although this study is rigidly designed, several limitations still exist. Firstly, the publishers may tend to publish those with positive results, which may have caused publication bias. Secondly, the study selects publications merely in English language, which may not cover those written in other languages. Thirdly, the library resources are limited, which may not include all the related publications.

### Future research directions

Blended learning may be promising especially during this COVID-19 pandemic time, and information technology scientists may focus on the development of more advanced and effective devices to improve blended learning effectiveness. Various factors such as perceived learning effectiveness, perceived usefulness and easiness, social presence, and demographic variables may be considered when we design blended learning technologies in the future. Future researchers could also enhance students' emotional intelligence and cognitive engagement, leading to favorable learning habits (Iqbal et al., 2022).

## Data availability statement

The original contributions presented in the study are included in the article/supplementary material, further inquiries can be directed to the corresponding author.

## Author contributions

ZY conceptualized, designed, collected, analyzed the data, wrote, edited, and polished this article. WX and PS revised, proofed, funded and edited the article. All authors contributed to the article and approved the submitted version.

## Funding

This work is supported by 2019 MOOC of Beijing Language and Culture University (MOOC201902) (Important) Introduction to Linguistics; Introduction to Linguistics of online and offline mixed courses in Beijing Language and Culture University in 2020; Special fund of Beijing Co-construction Project-Research and reform of the Undergraduate Teaching Reform and Innovation Project of Beijing higher education in 2020-innovative multilingual + excellent talent training system (202010032003); The research project of Graduate Students of Beijing Language and Culture University Xi Jinping: The Governance of China (SJTS202108).

## Conflict of interest

The authors declare that the research was conducted in the absence of any commercial or financial relationships that could be construed as a potential conflict of interest.

## Publisher's note

All claims expressed in this article are solely those of the authors and do not necessarily represent those of their affiliated organizations, or those of the publisher, the editors and the reviewers. Any product that may be evaluated in this article, or claim that may be made by its manufacturer, is not guaranteed or endorsed by the publisher.
